# LATIN-MH: a model for building research capacity within Latin America

**DOI:** 10.1017/gmh.2016.32

**Published:** 2017-01-26

**Authors:** B. B. Bonini, R. Araya, J. Quayle, M. Silva Evangelista, LeS. N. Price, P. R. Menezes

**Affiliations:** 1 Department of Preventive Medicine at the Faculty of Medicine, University of São Paulo, Sao Paulo 01246-903, Brazil; 2 Centre of Global Mental Health, London School of Hygiene and Tropical Medicine, London, UK; 3 Department of Health and Human Services, National Institute of Mental Health, National Institutes of Health, Washington, DC, USA

**Keywords:** Capacity building, Latin America, mental health, LATIN-MH

## Abstract

**Background.:**

Latin America Treatment and Innovation Network in Mental Health (LATIN-MH) is a research hub located in Brazil and Peru that conducts a research project to help reduce the treatment gap in mental health in Latin America (LA). Besides its research core, LATIN-MH has a Capacity Building (CB) component that aims to help young researchers receive the specific training to contribute to the growing scientific production in mental health in LA.

**Methods.:**

LATIN-MH proposal in CB includes a series of actions to prepare professionals in the research area. The main proposals are described here, which include online study groups, promotion of scientific meetings, hands-on training in different levels and sharing of information.

**Results.:**

LATIN-MH CB activities are at its initial stages but the proposed activities were well evaluated by the participants. The first participating fellows who finished their fellowships are contributing elsewhere in the mental treatment and human resources formation area.

**Conclusion.:**

The repercussion of LATIN-MH actions in CB and its evaluation, particularly on the formation of human resources and dissemination of information, show that the hub is contributing to the critic formation of young researchers and the circulation of important information.

## Background

Research in mental health in low-and-middle-income countries (LMIC) has been historically neglected. Although there has been a relative increase in research outputs, the situation remains critical (Bressa *et al*. 2005; Razzouk *et al*. 2006; Mari, 2014). There is little funding for research and few trained mental health researchers in LMIC, resulting in an unsurprisingly limited evidence base to inform mental health policy.

Psychiatric conditions represented 22.2% of the overall global burden of diseases in Latin America (LA) countries in 2002 (OPAS, 2009). Psychiatric disorders have been a growing condition in LMIC, where approximately 20% of the adults experience mental health issues during their lifes (Demyttenaere *et al*. 2004).

The World Health Survey showed that 18.8% of the people living in São Paulo, Brazil had received a diagnosis of depression in the last 12 months (Moussavi *et al*. 2007). Other studies show that 30% of the Brazilian adult population has experienced mental health disorders in their lives (Ludermir & Lewis, 2003; Lima *et al*. 2008).

In Lima, capital of Peru, it is estimated that 7% of the population is suffering from depressive symptoms, while 30% of the people suffer from mental health disorders (Loret de Mola *et al*. 2011).

The treatment of mental conditions around the world still faces huge challenges, from the lack of respect to the human rights of people with mental health problems to the deficit of allocated budgetary and human resources (Patel *et al*. 2011). To address the treatment gap in mental health it is important to think of ways of ‘delivering effective treatments in the real world’ which, in turn ‘depends crucially on the development of human resources’ (idem). It is not different in LMIC, where the lack of specialized personel to treat mental health problems is an issue of continental proportions.

Eaton *et al*. (2011) enphasize that in most LMIC there is such a disproportion beteween the existing qualified professionals and the people needing mental health care that ‘there is no prospect of psychiatrists being able to deliver the care that is needed in the foreseeable future’. Defending a new paradigm of public mental health these authors the importance of partnerships between research institutions, research and practionners in LMIC, based on local capacity building (CB) ‘to ensure high scientific standards and participation by all stakeholders, including people using mental health services’.

According to Kakuma *et al*. (2011), ‘the estimated total number of mental health care workers needed in the 58 countries of low and middle income in 2005 was 362 000, representing 22.3 workers per 100 000 people in low-income countries and 26.7 workers per 100 000 people in middle-income countries. This comprises 6% psychiatrists, 54% nurses in mental health settings and 41% psychosocial care providers’. The authors verified a shortage of 1.18 million mental health workers for all 144 LMIC. To address this shortage different strategies are proposed, including the use of task shifting and the education of mental health service providers.

In 2015, there were 9010 psychiatrists in Brazil, a ratio of 4.48 specialists per 100 000 inhabitants. Of those, 54% were concentrated in the southeast region of the country, which happens to be the richest region of Brazil. Only 2.1% of them are located in the north region of Brazil (Scheffer, 2015). The country counts with other mental health professionals, such as psychologists, mental health nurses and social workers, but these numbers show a shortage of professionals trained to work with mental health in the country. Similar scenario can be observed in Peru, where the mental professional ratio is 2.06 psychiatrists and six psychiatric nurses per 100 000 people in the country (Jacob *et al*. 2007).

It is essential to strengthen local capacity to retain trained researchers and to produce evidence-based knowledge to ground mental health policies. Brazil is one of the very few countries in the region that has developed active suppor programs to retain trained researchers. As a result, Brazil is standing out in terms of scientific output among LMIC. In 2008, publications by Brazilian researchers in peer-reviewed science journals represented 2.7% of global scientific publications in all areas of research, with a faster growth in public health sciences (UNESCO, 2010).

Mari (2014) points out that the scientific production in the field in Brazil is slowly growing, but that its impact is still small. On the other hand, psychiatrists are starting to show some interest in public health issues which, in turn, can result in better planning and implementation of public health policies for mental health and address the mental health treatment gap.

In 2010, the National Institute of Mental Health (NIMH), one of the units of the National Institutes of Health (NIH) from the USA, released a funding opportunity for LMIC to develop research in mental health, by creating the ‘Collaborative Hubs for International Research in Mental Health’. The project aimed to help the hubs not only to develop research in mental health, but also to become centers for developing research capacity for LMIC (Collins, 2010).

The Latin America Treatment and Innovation Network in Mental Health (LATIN-MH) is one of the five research hubs awarded by the NIMH to increase research capacity that can create better strategies to reduce the mental health treatment gap in LMIC. This hub is the result of a collaborative effort between the University of São Paulo (Brazil) and the Universidad Peruana Cayetano Heredia (Peru), counting with the support of the Northwestern University through ‘The Feinberg School of Medicine's Center for Behavioral Intervention Technologies (CBITs)’ and the ‘London School of Hygiene and Tropical Medicine’. It aims at conducting innovative public health-relevant mental health research in LA and build relevant research capacity in the region, collaborating with other regional hubs throughout the world.

The research component of LATIN-MH focus on developing and testing a low-intensity psychoeducational intervention delivered via smartphones for depressed people with a comorbid chronic medical condition (hypertension and/or diabetes) in Brazil and in Peru. The intervention is based on task shifting and counts on Nurse Assistants (NAs) and Nurses to motivate the participants’ and to monitor their performance.

The CB component of LATIN-MH is an initiative to increase mental health research capacity in LA. Its goal is to create an active network and to prepare researchers to generate and disseminate evidence to guide policy makers to implement strategies to reduce the mental health treatment gap in the region, so that more effective interventions can scale up.

LATIN-MH is organized in different committees: Executive Leadership Committee (ELC); Capacity Building (CB); Administrative Core (AC); RCT Steering Committee (RCT SC); Data Coordination Center. Specific Working Teams (WG) are created as necessary to face the challenges and needs of each phase of the study.

From the CB perspective, LATIN-MH aims, in this context, to develop effective and efficient programs that can train researchers from poor resource settings and create a net in mental health to provide innovative proposals and solutions for chronic problems in public mental health management.

In order to do so, LATIN-MH CB, which is still in its initial stage, is developing a series of activities to help young researchers to better develop research in LMIC.

## LATIN-MH CB proposal

One of the main worries of the CB effort of LATIN-MH was to develop activities that could reach a large number of young reserchers who, otherwise, would not have the possibility of interacting, discussing and improving their research skills.

With the recognition that mental health has to be addressed on a global scale, different efforts were developed to improve not only ‘evidence-based, culturally appropriate, cost effective, feasible care’ worldwide, but also to direct attention on building mental health capacity in LMIC (Thornicroft *et al*. 2012, p.14).

Fricchione *et al*. (2012) proposed a strategy for CB. They pointed out that besides the establishment of partnerships to improve clinical training and certification of mental health professionals, the development of research units ‘should be part of the center of excellence, so that sustainable research training can be developed within LMIC’. This is fundamental to create scientifically and culturally significant.evidence-based knowledge.

In 2004 the Global Forum for Mental Research, in collaboration with WHO, mapped research capacity in LMIC in order to raise awareness of the need for strengthening research capacity regarding mental health (Thornicroft *et al*. 2012, p. 14). The results showed the urgency of initiatives to increase ‘research capacity, includind skills in epidemiological and/or public health methods, knowledge translation and exchange, leadership, mentorship and advocacy’.

Some models for the development of research capacity have been proposed and tested with the funding of international agencies, such as the UK Department for International Development Research Programme Consortium, the WHO Aliance for Health Policy and Suystems Research and the Wellcome Trust.

Based on the experience of the ‘Building Research Capacity in Sub-Saharan Africa in Mental Health and Poverty Project’ (NHaPP), Thornicroft *et al*. (2012) enphasizsed the importance of some and goals:

- give training to both junior and senior investigators;
- use different methods for intercountry communication;
- stimulate site visits;
- identify a person in each site to be responsible for CB development;
- adopt a ‘train the trainer’ approach;
- implement an online journal club to discuss the key scientific production;
- identify possible contributions of each partner involved;
- propose activities to implement skills of different levels investigators;
- offer opportunities for junior staff to practice key skills.

These guidelines were used as reference when LATIN-MH planned its CB actions. The proposed ‘stages of research capacity development’ of Thornicroft *et al*. (2012) were also considered. These stages are: (1) Introduction to Research; (2) Initial Familiarization with the work of research teams; (3) Attendance at short courses on specific research themes; (4) Master level programs; (5) Predoctoral preparatory Fellowship and doctoral fellowships; and (6) Postdoctoral research positions and opportunities. Due to specific conditions, the initial experience of LATIN-MH focused on items 1–3 and 6, constituting a four-level reference structure, which could be expanded in due course. These steps developed in LATIN-MH hub, are described below in Table 1 and related to the goals appraised from the NHaPP experience (a to h).

**Table 1.** Planned steps for LATIN-MH CB and its relationship with goals, activities and target investigators

LATIN-MH CB proposals	Main related goals and strategies	Programs and activities
Introduction to research	a; b; c; e; g; h; i	Study Groups
		RA training
		Fellow trainings
		Online courses
Familiarization with the work of research teams	a; b; c; e; g; h; i	Hands on training
		Working groups
Short courses on specific research themes	a; b; h; i	Symposia
		Events
		Meetings
Postdoctoral research positions and opportunities	a; d; e; h	Fellowships

The specific actions related to the atages are described next.

### Introduction to research

Introduction to research is a goal porsuited through different activities, some as part of the regular team meetings, some as more formal activities. Here the focus will be on structured activities proposed to attain these goals.

#### Online study groups

To help researchers and other professionals interested in mental health research, LATIN-MH developed a series of online meetings denominated ‘study groups’. These groups aim at introducing and discussing practical and theoretical research aspects. Anyone interested in research is invited and can join the online sessions. Participation is free of cost, but requires previous registration online.

##### Modus operandi

The LATIN-MH study group gathers once a month for webinars to discuss topics of interest for researchers in the field. Led by a senior investigator, the webinars have 1-h duration and are open to anyone interested in the subject. The groups are held in Portuguese, English and Spanish, alternatively and the language of the meeting changes accordingly to the guest speaker who leads the discussion.

Those who register for the group have granted access to the LATIN-MH Moodle platform, where the material to be discussed in the meeting is available – usually a paper or a slide presentation. In this platform, there is also a ‘discussion forum’, other subsidiary literature and material from previous meetings. Each meeting is recorded and made available in the LATIN-MH YouTube page.

From April to August 2016 there were five study groups held under this format. The main features related to these groups are presented on Table 2.

**Table 2.** Summary of Study Group activities

Theme/Guest speaker	Language	Month	Attendance (registered/present)	Level of education	Origin^a^
Depression in the Amazon Paulo Menezes	Portuguese	April	49/21	Undergraduate: 4.9% Graduate: 47.6% Master's student: 9.5% Master degree: 9.5% Ph.D.: 19% Other: 9.5%	46 Brazilians Two Peruvians One African
Implementing Research Jaime Miranda	Spanish	May	53/24?	Undergraduate: 16.7% Graduate: 41.7% Master's student: 4.1% Master: 16.7% Ph.D.: 12.5% not informed: 8.3%	? 22 Brazilians 39 Peruvians Two Americans
Task Shifting Ricardo Araya	English	June	34/21	Undergraduate: 10% Graduate: 60% Master's student: 10% Master: 10% Ph.D. student: 5% Ph.D.: 5%	25 Brazilians Six Peruvians One Colombian One Ghanian
Data Management Heloisa Claro	Portuguese	July	36/29	Undergraduate: 11.5% Graduate: 50% Master: 15.4% Ph.D. student: 3.8% Ph.D.: 3.8% Other: 15.4%	33 Brazilians Three other nationalities
Mental Health Reform in Peru Humberto Castillo	Spanish	August	45/32	Undergraduate Graduate Master's student Master: Ph.D. student: Other:	27 Brazilians 16 Peruvians One American One Argentine

a Based on the identification of participants.

The online sessions have been quite dynamic and spontaneous. They provided the chance of exchanging experiences, posing and solving doubts with experts, amplifying and deepening the discussion on some important issues on mental health research with people from different countries, scenarios and background. The Adobe plataform provided the necessary structure for the activity and the fact that the material is made publicly available through youtube was dfeeply appreciated.

The acceptance and evaluation of this activity is done online by sending a small questionnaire along with the certificate of participation. Only a few people have given feedback and grades to the activity, but among those who did, all grades were above 8. The fact that many participants return for the following study groups after a first participation suggests it is useful and enjoyed.

#### Online courses

Another proposed activity is the ‘LATIN-MH Online Courses’. Differently from the open proposal of the study groups, the online courses aim at offering opportunities of learning basic and necessary information on research methodological and strategic issues.

We are developing three distance-learning courses to help young researchers develop their research skills and conduct their own research. For the next few years, the hub will make available introductory courses in Epidemiology, Mental Health research and how to build a research project. The online courses are a joint effort between the senior investigators of the hub and the post-doctoral fellows. The courses will be mandatory for all the hub's fellows and will be open to the general public. It is important to mention that, since the hub itself is a result of an international effort, the courses will be available in the three main languages of the hub: Portuguese, English and Spanish, whenever possible.

### Familiarization with the Work of Research Teams

The main strategy to attract professionals interested in research issues and to improve hub's member skills is the ‘hands on’ approach offered in different levels through the CB proposal. It includes activities directed to core staff of the research team as well as specific programs of training to selected personnel.

#### ‘Hands on’ training

##### The working groups

One of LATIN-MH CB main goals is to provide ‘hands-on’ training for researchers in all levels of expertise. In order to accomplish that, all of its members and fellows areencouraged not only to participate in theoretical discussions, but also to develop research of their own and/or to work within the main research frame component of the hub's project.

LATIN-MH has two site teams, one in São Paulo, Brazil and another one in Lima, Peru. However, the members of both sites could work together in the different committees and working groups of the hub, via e-mails and Skype meetings toward the construction of the research documents and protocols. These working groups happened under the supervision of a senior member (PI or CoPI of the study). The proposal was that people with different expertise would participate on the ellaboraion of the documentation and strategy of the research project, so everybody could profit from the experience. At the same time, LATIN-MH would come up with a product which was, at the same time, the result of a collective effort and high quality. This model proved very efficient, although it requires availability of all people involved to spend time and effort in this kind of construction

Employing this strategy, we had the following working groups during the preparation phase of the RCT:

- Protocol working group: responsible for reviewing pilot study clinical data and material and proposing and writing the protocol for São Paulo and Lima study, including forms and procedures, to be analysed by the PIs and the LATIN-MH Steering Committee.
- CONEMO working group: responsible for reviewing pilot study application data and material, organizing and consolidating the sequence and content of the participant app, as well as the professional interfaces for monitoring participants’ performance, under the orientation of a PI and the Steering Committee.
- Data center working group: responsible proposing an efficient database structure for both sites, based on the protocol instruments and forms, compliant with ethical guidelines and respect for human subjects’ rights, as well as a hierarchy of access and reports of different member of LATIN-MH.
- Intervention working group: responsible for reviewing pilot study intervention and supervision data and material and proposing to the Steering Committee a final model of intervention using the app CONEMO.

##### LATIN-MH fellowship

Beyound group activities, there are other types of ‘hands on’ training in LATIN-MH. Hub fellolws usually develop many activities under this label. The hub has a fellowship program, which invites fellows from diferent fields or areas. The program counts with different types of fellowships in other to gather people with different skills that can contribute in specific ways to the hub's research.

There are three categories of fellows in LATIN-MH: Post Doctoral Fellows, Junior Fellows and Research Fellows.

Four *post-doctorate fellows* are responsible for developing proposals and/or supervising activities in specific areas of the project: Capacity Building, Data Center, Intervention and Project Coordination. In this scenario, these fellows have the opportunity of employing their knowledge and expertise in their daily chores, while they can improve their research thinking and deal with usual field planning and trial conduction challenges. There is no restriction for the number of fellows under this category.

Post Doctoral fellows’ program lasts 12 months and can be renewed once, for the same length of time. Until 2015, the hub had two other postdoctoral fellows, comprising a total of six postdoctoral researchers who helped and benefited from the participation in the research activities of LATIN-MH up to 2016.

The hub also counted on two *junior fellows*, one from Colombia and another from Guatemala. These fellows had their work related to hub's main goals, but were not directly involved in the primary research activities of the project. There is no core curriculum for these fellows. They are invited to participate in the regular activities of the hub, under the supervision of a PI. Their activities are mostly provided by the institutions where they are allocated. However, junior fellows have to complete some mandatory activities, such as the online courses provided by the hub.

The mentorship provided to these junior fellows was a personalized support for the development of their careers. They received joint mentorship from a local and an international senior researcher involved with the hub's activities. It is important to mention that the hub does not fund junior fellows. However, they received support for preparing their own grant applications and they could join specific training programs from their mentors’ institutions. Writing grants and applying for funding is an essential task for any successful researcher, and we aimed to introduce this skill early in their careers.

The *RCT fellows* (Research Fellows) are young researchers invited to work in the main research component. They are selected among young researchers who apply to participate in the research core of the project and show interest in academic work.

RCT fellows receive ‘hands-on training’ occurring previously and during the randomized trial that will happen in Brazil and Peru There are five Research Fellows in the hub and they develop their daily activities under the supervision of post doctorate fellows or hired personnel. These fellows (three in the São Paulo site and two in the Lima site) are funded by the hub and are encouraged to prepare their own applications for their formal postgraduate training and to participate in the hub's publications.

The main activities of the RCT fellows are: to participate in the LATIN-MH training activities and specific site team meetings; to take the NIMH Good Clinical Practices course; to participate in the research assistants’ (RA) training sessions; to participate in the mentorship meetings to develop a research project for graduate program of their choosing; and to help in the preparation of publications from the hub.

This program is innovative because it prepares researchers to become effective trainers themselves. All too often, it is assumed that researchers are able to train others effectively, even though they have never received training themselves nor prepared to do so. We provide our RCT fellows with the basic tools to do this job properly.

##### Training of the RAs and NAs

RAs’ training

The selection and training of the 20 RAs for the trial data collection took place in several steps.

1. The production of all material needed for the training (Manual of the RA; presentations; evaluation material);
2. The installation of the database apps in the tablets (data collection app; manuals; forms);
3. Dissemination of the RA vacancies for the project;
4. Selection of candidates (first using academic and experience criteria and then through a hands on training/selection model);
5. Hiring of the 20 selected candidates and beginning of the field-training course.

In this 120-h intensive capacitation course, RAs got in contact with theoretical and practical aspects of their role and tasks to be developed, the project itself, visits to the field, forms and procedures, as well as the ethical aspects involved in research with human subjects. Considering language difficulties (most are not fluent in English), the material offered by NIH was employed in group sessions guided by facilitators in order to cover all four units of the online course.

The selection and training process started with 108 candidates and was planned so that, beyond selecting the candidates by their theoretical worthiness, research practice and habilities were also taken into account. In the initial phase, conducted with 96 candidates, they were observed and evaluated daily in order to assess *how much the candidate to RA could absorb what was being taught and if the candidate was suitable for the field work*. Besides theoretical classes, different strategies were employed in this training, including role-playing, visits to the field, manipulation

The Nurse and NAs Training

LATIN-MH research project aims at developing and testing an intervention based on task shifiting to treat depressive symptoms in people with hypertension and diabetes. It proposes the use of an applicative based on behavior activation and delivered by smartphones. The professionals responsible for monitoring participants’ activities and performance are nurses and NAs (in Peru and São Paulo, respectively), in a task-sharing approach. To accomplish these tasks these professionals have to be selected and trained. Here we will briefly describe the training of the NAs from the 20 primary care health units who participate in the research project in São Paulo site that already took place.

The selection and training of NAs happened according to the project's needs and the health units’ possibilities. The training took place in the facilities of the institution responsible for the management of the health units in a partnership with the city health system. The training initial sessions lasted 8 h, and the NAs were divided into two training days.

The training was provided by the two clinical psychologists who respond for the intervention clinical supervision during the trial. Before the training, the necessary material was prepared and the apps were installed in the smartphones and in the tablets. The training involved not only grounding theoretical aspects of depression associated with chronic diseases and behavior activation, but also practical training with the electronic devices and role-playing. Beyound this, routine supervision provides an important complementation to the training.

#### Other specific training

As part of the routine training, all LATIN-MH team members go through clinical research courses training, particularly those involving ethical aspects of clinical research, such as the ‘Good Clinical Practices’ from the National Institute of Allergy and Infectious Diseases, from the USA NIH.

Besides, all members are encouraged to participate in all of the hubs’ CB training activities that are not specific for a certain group, as well as in research-related open activities of the institutions connected to the hub.

### Short courses, scientific events and meetings

#### Hubs meetings

LATIN-MH members have participated of events and meetings in order to favour discussion on mental health issues and help prepare the trial proposal. These meetings sometimes involved other collaborative hubs worldwide and LATIN-MH hub members, alternatively.

##### LATIN-MH meetings

From the beginning of the hub's activities, there was an effort to produce high quality collective work based on evidence, as well as to provide CB opportunities to junior members, trainees and fellows. Aligned to this guideline, the goals of these intrahub meetings referred meanly to the planning and the implementation of the pilot study and the RCT while discussing other hubs’ and researchers’ proposals, experiences and solutions for common problems related to the use of task shifting in mental health treatment.

In the first year (2013), the LATIN-MH members met twice: once in July, in Peru, for the assessment of the Peruvian scenario for the beginning of the pilot and later in São Paulo for the LATIN-MH first summit. When the NA role in the project was discussed and the NA Support System was presented in the realm of the intervention

In 2014, the hub had some opportunities to get the team together to further discuss the issues regarding the pilot studies in Brazil and Lima. There were also some visits made by some of the team's members for training periods.

In 2015, before the beginning of the pilot fieldwork, both sites had the visit of a northwestern representative to assess the usability of the technology and final alignment of the system. In June 2015, after the conduction of the pilot studies, the hub met for the third time, bringing all members for a 3-day meeting to discuss the evolution of the project, the results of the pilots and to prepare the trials.

##### Interhubs annual meetings

The first participation of a LATIN-MH representative in these meetings was in 2013, in the South Africa meeting held in Cape Town. In October of 2014, three members had the opportunity to visit New Delhi, India, for the Interhubs annual meeting, in order to share the work that was being done in each hub, present LATIN-MH proposaland discuss the issues and implications of the projects. In 2015, nine members of the hub went to Ghana for the interhubs annual meeting held in Accra. This opportunity was also employed to further discuss the implementation of the RCT.

São Paulo hosted the 2016 Interhubs’ in São Paulo in September, and received 60 representatives of all the hubs for a very proficuous meeting, where hubs in different stages of their research and CB proposals could exchange experiences and discuss the implications of their work.

The annual meetings provide exchange of information and expertise, helping young researchers to better understand how to conduct research in the real world. They also allow them to meet experienced researchers and to delevop their own research projects.

##### Other exchange and training opportunities

In June of 2014, two of our team members attended the “*Solving the grand challenges in global mental health: partnerships for research and practice*” workshop, promoted by NIMH, in Maryland – DC. In 2015, during the Interhubs Annual Meeting, LATIN-MH members could participate in the workshop on ‘Cost-effectiveness Assessments across and within the Hubs’, which provde tools for preparing the hub's proposal on this aspect.

During the World Health Organization and the World Bank Meeting “*Out of the Shadows: making mental health a global priority*”, one of the LATIN-MH PIs, Dr. Paulo Menezes, was invited to join the workshop about “*Mental Health Innovation in the Americas: Closing the Treatment Gap*” promoted by the NIMH and the Grand Challenges Canada. During this workshop, he presented the LATIN-MH efforts to reduce the treatment gap in Brazil and Peru.

One member of the LATIN-MH team in Peru, Francisco Diez-Conseco, applied for and received a grant from the *Global Mental Health Research Training Institute* for a training course in Bethesda, Maryland, from June 6–9, 2916, in the US NIH. This short-term, scenario-based training aimed at promoting multidisciplinary research, implementation science and novel research methodologies to a cohort of researchers from diverse fields who will help to lay the groundwork for future Mental Health research projects and begin to build the evidence base for impactful technologies.

#### The symposiums

The symposiums constitute a formal moment for researchers’ training and information. The only requirements to attend the symposium are the ability to understand English, due to the fact that the activities are held in English, and previous registration. The activities were free of charge.

The first Latin America Treatment and Innovations Network Symposium took place on June 2015, at the Faculty of Medicine of the University of São Paulo, with the support Department of Preventive Medicine. This activity aimed at involving anyone with an interest in global mental health research and preparing young researchers to carry their own studies in mental health.

The symposium was planned to offer two different moments. In the morning, there was a panel to discuss “*Integrating mental health into general health care*”, with the presentation of different scenarios influencing mental health trreatment (São Paulo, Lima and London). The morning activity was closed by the presentation of funding possibilities for research in mental health. In the afternoon, there were two workshops. The first, “*Mobile Technological Interventions in Global Mental Health*”, was associated with LATIN-MH experience in developing applicatives to be used to address depressive symptoms related to chronic diseases. The other addressed “*RCT designs and methodological issues in Global Mental Health*” and besides a review of modles and guidelines allowed for the discussion of the audience's doubts and concerns towards conducting RCTs.

Senior hub's researchers were responsible for the activities in both morning and afternoon activities. There were 52 applicants for the activities and 35 participants. The applicants were, mostly, from São Paulo city and state, but there were people from other states in Brazil and from Lima, Peru. Most of the attendees were at a doctoral degree level (they either concluded or were in the process of it), followed by post-doctoral and master's degree level participants.

In September 2016, the *Second Latin America Treatment and Innovations Network Symposium* took place to discuss the theme ‘Increasing Research Capacity’. This time two workshops were planned: Practical aspects of international opportunities grant in mental health research”, and ‘Conducting field trials in mental health’. In the first one, there were 27 attendees and 24 people participated in the second.

The overall evaluation of both symposiums was very good and attendees reinforced the impotance of having this opportunity to discuss issues related to field trials with peers and experts. On the other hand, it is important to consider the relatively low attendance rates of these events. Brazil's mother language is Portuguese and not many Brazilians are familiar with English may be associated with the relatively low number of sympoiums’ attendees. Another aspect to take into consideration is that there were no fees involved, which favours the application without the compromise to be present.

### Dissemination of information

One of the key aspects of developing research is to circulate information and results that can ground professional therapeutic decisions and further research developments. LATIN-MH invests not only in the production of scientific reports, but also in the dissemination in the social media, to amplify its reach to the other researchers and health professionals, as well as to reach the non-professional public.

The hub created a website (http://www.latinmh.com.br) in three different languages (Portuguese, Spanish and English) to facilitate the spread of information and activities. The website is also a repository for articles of interests, training manuals, research protocols, questionnaires and other quality assurance methods. There is an emphasis on the use of technology in mental health care and new information on behavioral intervention technologies and task shifting. We would like library to become the reference source of information on mental health research in LA. To further disseminate all of the hub's CB activities, we have a biannual newsletter, also available in the site, summarizing information about the hub's progress, activities and opportunities.

To connect with other researchers and mental health professionals, quickly sharing information and articles of interest, the hub maintains a Facebook page (http://www.facebook.com/latinmh) created on September 2015. Open to anyone who wishes to share content and discuss information, this page also aims at spreading information to lay population. The fans of the page are from different locations: Brazil, Peru, Ghana, the USA, Finland, the UK, France and Costa Rica. The page is updated every 2 days, having a post average of three posts a week.

We also maintain a Twitter account (https://twitter.com/latin_mh) created on February of 2016 to help spread information across social media. The LATIN-MH Twitter account was. It has 11 followers and follows 15 Twitter accounts. It currently has 52 tweets, and the same average post of tweet as the Facebook's.

LATIN-MH has also developed a library of recorded lectures and seminars using online platforms to store online courses and content. The LATIN-MH YouTube channel contains the lectures of the LATIN-MH Symposium and the videos of the study groups. All courses and seminars promoted by the hub are recorded and the videos are available in the YouTube channel and all presentations are available for download at the website. This library is a good way to help young researchers develop their own projects in the field. It is also a way to disseminate information to lay people.

LATIN-MH participated in the ‘Innovation Fair’, that happened on April 13th 2016, in Washington-DC, during the World Health Organization and the World Bank Meeting “*Out of the Shadows: making mental health a global priority*”. The Hub's proposal and achievements were presented and it was a great opportunity for disseminating ways of addressing task shifting as a possibility of addressing the treatment gap in LMIC.

## Conclusion

The LATIN-MH CB effort, although still in its initial stages, aims to help amplify mental health research effort in LA in order to address the mental health treatment gap in the region. The focus is on the dissemination of information and the ‘hands-on’ training of researchers. With its unique international composition, the hub is building a network of young and senior researchers to address the gap in mental health treatment in the region. By including young researchers in key positions in such a large multi-centric study, we hope to empower them to conduct, in the near future, their own research work.

The focus on online and multi-language activities aims to reach a bigger number of young researchers in LA, especially the ones with few nearby of resources count on when developing their own projects.

Altough most CB activities in LATIN-MH are recent, they have positively influenced the research activities of the hub, either because of the research and NAs or as a result of the post doctorate fellows. The hands on training proved to be an important way of developing skills and teaching habilities. The working groups brought togetther groups of professionals and trainnees form different background and different countries to work together on specific tasks and put together a strong research project and monitor its development. The first participating fellows who finished their fellowships are already contributing elsewere in the mental treatment and human resources formation area. Other activities, open to the general research community, were well evaluated and hopefully will show a qualitative and quantitative impact in the scientific production on mental health research and treatment in LA. The repercussion of LATIN-MH actions in CB and its evaluation, particularly on the formation of human resources and dissemination of information, shows that the hub is contributing to the critic formation of young researchers and the circulation of important information.
